# Pseudomonas aeruginosa Induces Interferon-β Production to Promote Intracellular Survival

**DOI:** 10.1128/spectrum.01550-22

**Published:** 2022-10-03

**Authors:** Ling Yang, Yu-Wei Zhang, Yang Liu, Ying-Zhou Xie, Dong Weng, Bao-Xue Ge, Hai-Peng Liu, Jin-Fu Xu

**Affiliations:** a Department of Respiratory and Critical Care Medicine, Shanghai Pulmonary Hospital, School of Medicine, Tongji University, Shanghai, China; b Institute of Respiratory Medicine, School of Medicine, Tongji University, Shanghai, China; c Clinical Translation Research Center, Shanghai Pulmonary Hospital, School of Medicine, Tongji University, Shanghai, China; Peking University People’s Hospital

**Keywords:** *Pseudomonas aeruginosa*, IL-1β, cGAMP, AKT, IFN-β, AKT signaling

## Abstract

Pseudomonas aeruginosa (PA) is known as one kind of extracellular pathogens. However, more evidence showed that PA encounters the intracellular environment in different mammalian cell types. Little is known of innate immune factors modulating intracellular PA survival. In the present study, we proposed that interferon-β (IFN-β) is beneficial to the survival of PA in the cytoplasm of macrophages. Furthermore, we found that interleukin-1β (IL-1β) induced by PA suppresses IFN-β response driven by the cGAS-STING-TBK1 pathway. Mechanistically, IL-1β decreased the production of cyclic GMP-AMP (cGAMP) by activating AKT kinase. cGAMP is necessarily sufficient to stimulate the transcription of IFN-β via the STING adaptor-TBK1 kinase-IRF3 transcription factor axis. Thus, our findings uncovered a novel module for PA intracellular survival involving IFN-β production restricted by IL-1β and provided a strong rationale for a potential clinical strategy against pulmonary PA infection patients.

**IMPORTANCE** The link between innate immunity and intracellular Pseudomonas aeruginosa is unclear. Our studies illuminated the role of interferon-β (IFN-β) in remote intracellular PA infection. Furthermore, our experimental evidence also indicated that IL-1β is a negative regulator of IFN-β production and, in particular, P. aeruginosa infection. The inhibition of IFN-β may be used as a potential therapeutic method against pulmonary PA infection.

## INTRODUCTION

Pseudomonas aeruginosa (PA) is a conditionally pathogenic Gram-negative bacillus that connected with severe lung destruction and reduced lung function (1, 2). In China, the isolation of PA from adult bronchiectasis patients is a significant prognostic indicator (3). Traditionally, PA was defined as extracellular pathogen, but in fact, recent data have emphasized that several extracellular pathogens can enter host cells, resulting in a phase of intracellular residence. Some researchers have proposed that during the acute infection period of PA, the airway epithelial cells could engulf the bacteria into the cells before the biofilm is formed (4, 5). In addition, others have cocultured PA and epithelial cells to show the specific process of bacteria entering the cell (6, 7). An in-depth study has visualized the fate of PA within cultured macrophages, revealing macrophage lysis driven by intracellular PA (8). A study that traced the PA inside epithelial cells now makes it clear to researchers that PA is not simply an extracellular pathogen (9). To this end, revealing the immune escape mechanism of intracellular PA may provide new ideas for combating pulmonary PA infection.

Innate immune is the first line of defense against pathogen infection, and pattern-recognition receptors (PRRs) activate this process by recognizing pathogens (10). In response to infection, the activation of PRR upregulates relevant signaling pathways that enhance the host’s immune response and reduce microbial invasion. In this process, the inflammasome is a protein complex whose main function is to activate the function of caspase-1. The activated caspase-1 cleaves the precursor of interleukin-18 (pro-IL-18) and the precursor of interleukin-1β (pro-IL-1β) to produce mature IL-18 and IL-1β that will be released outside the cell, thereby amplifying the preinflammatory response and achieving the purpose of resisting pathogen invasion (11). The early response of PA infection in the lung is the release of the proinflammatory cytokine IL-1β (12, 13). The known inflammasome complex mainly includes NLRP1, NLRP2, NLRP3, NLRC4, and AIM2.

The innate immune system plays an important role in sensing various microorganism infections. A key surveillance mechanism is the cyclic GMP-AMP (cGAMP) synthase (cGAS) pathway. Briefly, the detection of DNA by cGAS leads to the synthesis of cGAMP, a strong activator of the STING-TBK1-interferon-β (IFN-β) axis (14, 15). It is worth mentioning that the sustained production of type I interferons is detrimental to the host, with intracellular bacterial infections such as Francisella novicida, Mycobacterium tuberculosis, and Listeria monocytogenes (16–18). The results implicated type I IFN-associated suppression of type 17 immunity and antimicrobial peptide production were the mechanisms for susceptibility to both Escherichia coli and Staphylococcus aureus coinfection during influenza infection (19). The role of IFN-β on the PA infection is controversial and urgently needs to be explored.

The serine/threonine kinase AKT, also known as protein kinase B (PKB), is a central node in cell signaling downstream of growth factors, cytokines, and other cellular stimuli. In particular, AKT-mediated phosphorylation of different proteins leads to their activation or inhibition. Regulation of these substrates by AKT contributes to activation of the various cellular processes, including survival, growth, proliferation, glucose uptake, metabolism, and angiogenesis (20). Indeed, AKT regulated the IFN pathway. The data from Kaur et al. provided definitive evidence that the AKT pathway is essential for the induction of IFN responses (21).

However, how these innate immune pathways regulate the survival of intracellular PA is poorly defined. Here, we demonstrate that IFN-β is beneficial to the survival of intracellular PA. Furthermore, IL-1β activated by the NLRP3 and NLRC4 inflammasome complex suppresses the IFN-β produced by the cGAS-STING-TBK1 pathway. Consistent with this finding, NLRP3 knockout mice and NLRC4 knockout mice displayed enhanced IFN-β production after PA infection. Mechanistically, IL-1β decreased the production of cGAMP by activating AKT kinase. The inhibition of IFN-β signaling to control the survival of the intracellular PA. In summary, this study uncovers a previously unrecognized key role for IL-1β in restraining IFN-β responses to restrict the survival of intracellular PA.

## RESULTS

### IFN-β promotes the survival of PA in the cytoplasm of macrophages.

**FIG 1:**
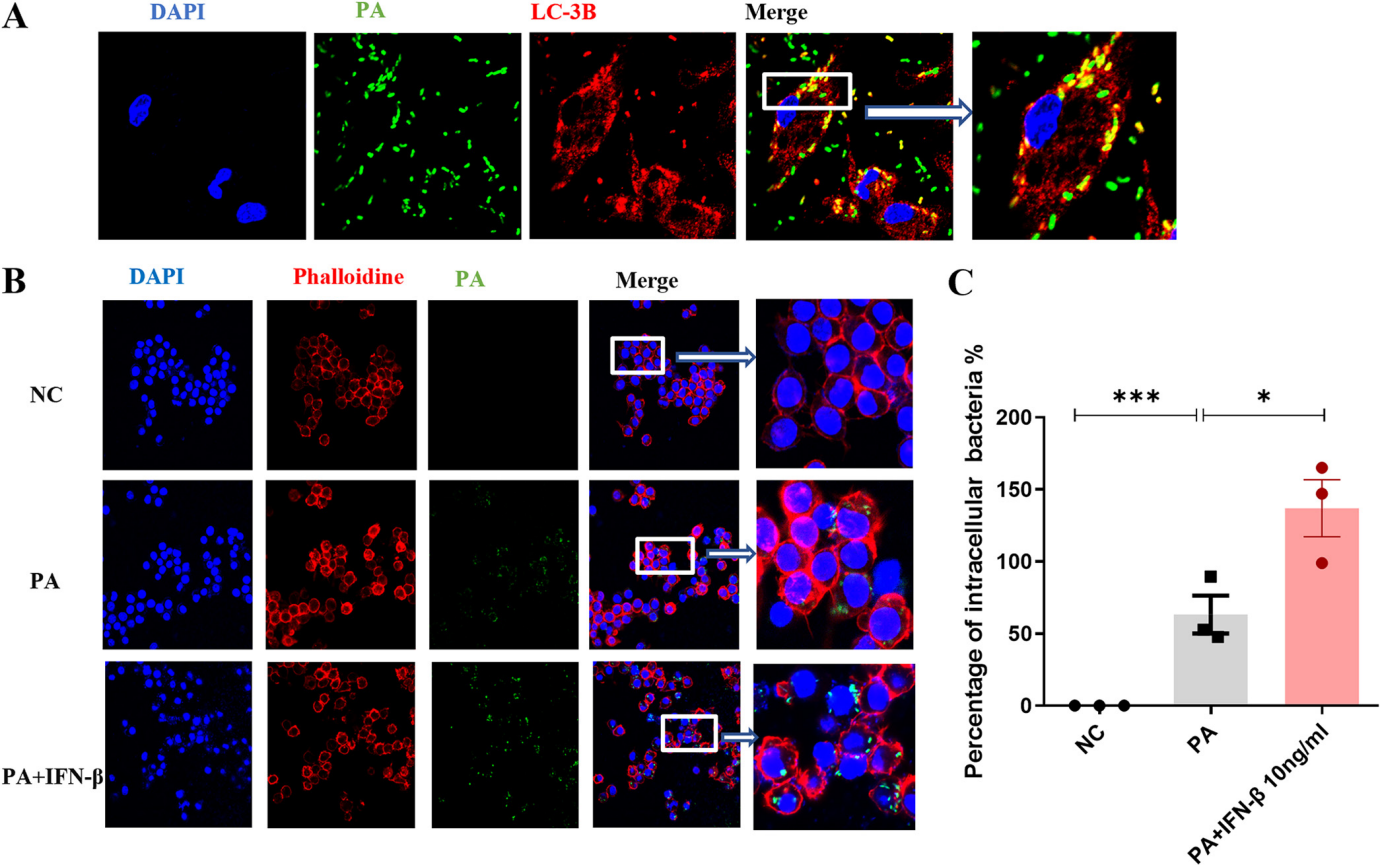
Interferon-β (IFN-β) promoted the survival of P. aeruginosa (PA) in the cytoplasm of macrophages. (A) The fate of PA in the mouse peritoneal macrophages cytoplasm with green fluorescent protein (GFP)-labeled PA (multiplicity of infection [MOI] = 10) infection for 2 h. (B) Pretreatment with IFN-β (10 ng/mL) or phosphate-buffered saline (PBS) as a control for 3 h and the intracellular PA in RAW264.7 cells with GFP-labeled PA (MOI = 10) infection for 2 h. (C) Pretreatment with IFN-β (10 ng/mL) or PBS as a control for 3 h and the percentage of intracellular PA of RAW264.7 cells with PA (MOI = 0.1) infection for 1 h (*n* ≥ 3). All experiments were performed three times. *, *P < *0.05; **, *P < *0.01; ***, *P < *0.001; ****, *P < *0.0001; DAPI, 4′,6-diamidino-2-phenylindole.

Pathogens are typically targeted by host cells to the autophagy-lysosome pathway as a strategy to inhibit their replication and limit infection (22). Thus, they have evolved strategies to evade or inhibit autophagy in order to persist in host cells (23). During lung infection by PA, autophagy plays an important role in mast cells and alveolar macrophages in the clearance of the bacteria (24). Moreover, PA survives in epithelia by ExoS-mediated inhibition of autophagy (25). Similar to the results of the above studies, we found that PA labeled with green fluorescent protein (GFP) could enter into macrophages and colocate with autophagy marker LC-3B (Fig. 1A). However, we also noticed that not all bacteria could be labeled by LC-3B to achieve lysosomal degradation (Fig. 1A). This means that PA can escape such clearance and persist inside the macrophages. To explore the role of IFN-β on the survival of PA in macrophages, we pretreated RAW264.7 cells with synthetic IFN-β for 3 h. Fig. 1B and C show that IFN-β promotes the survival of PA in the cytoplasm of macrophages.

### PA induces the production of IFN-β though cGAS-STING-TBK1 pathway.

**FIG 2:**
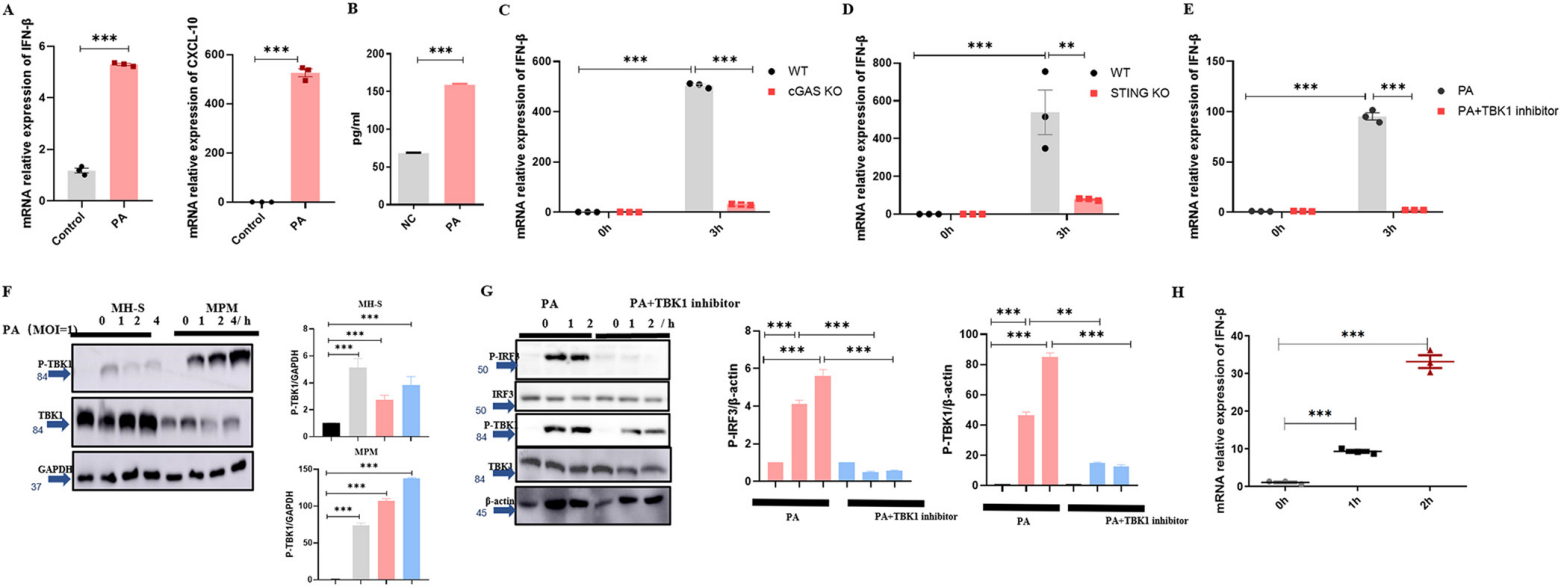
PA induced the production of IFN-β though the cGAS-STING-TBK1 pathway. (A) The mRNA relative expression of IFN-β and CXCL-10 of indicated mouse lungs with or without PA (3 × 10^6^ CFU) infection for 24 h (*n* ≥ 3). (B) The level of IFN-β in indicated lung homogenate with or without PA (3 × 10^6^ CFU) infection for 24 h (*n* ≥ 3). (C) The mRNA relative expression of IFN-β of WT and cGAS KO mouse peritoneal macrophages with PA (MOI = 1) infection (*n* ≥ 3). (D) The mRNA relative expression of IFN-β of wild-type (WT) and STING knockout (KO) mouse peritoneal macrophages (MPMs) with PA (MOI = 1) infection (*n* ≥ 3). (E) Pretreatment with TBK1 inhibitor or DMSO as a control and the mRNA relative expression of IFN-β of mouse peritoneal macrophages with PA (MOI = 1) infection (*n* ≥ 3). (F) Immunoblotting for total TBK1, P-TBK1, and glyceraldehyde-3-phosphate dehydrogenase (GAPDH) of MH-S cells and mouse peritoneal macrophages with PA (MOI = 1) infection (*n* ≥ 3). (G) Pretreatment with TBK1 inhibitor or DMSO as a control and immunoblotting for total TBK1, P-TBK1, P-IRF3, and GAPDH of mouse peritoneal macrophages with PA (MOI = 10) infection for indicated time (*n* ≥ 3). (H) The mRNA relative expression of IFN-β of MH-S cells with the DNA of PA (2.0 ng/μL) (*n* ≥ 3). All experiments were performed three times. *, *P < *0.05; **, *P < *0.01; ***, *P < *0.001; ****, *P < *0.0001.

IFN-β is an important part of innate immune in anti-virus. Recently, respectable studies have suggested that bacterial infection can also cause high expression of IFN-β (26, 27). To characterize IFN-β expression during PA infection, we used female C57/B6 mice to construct the model of acute pulmonary PA infection. The expression of IFN-β and CXCL-10 (Fig. 2A) increased as predicted. The level of IFN-β protein (Fig. 2B) in the indicated lung homogenate showed the same trend, and it was clearly observed that PA induced TBK1 phosphorylation (Fig. 2F). Next, we examined whether PA causes IFN-β production via the cGAS-STING-TBK1 axis. cGAS KO and STING KO mouse peritoneal macrophages were used to detect the expression of IFN-β. In the cGAS KO and STING KO group, the expression of IFN-β decreased significantly (Fig. 2C and D). At the same time, TBK1 inhibitor also decreased IFN-β expression, TBK1 phosphorylation, and IRF3 phosphorylation (Fig. 2E and G). In addition, we found the expression of IFN-β (Fig. 2H) increased in a time-dependent manner after the addition of DNA (extracted from PA), which further illustrated that PA causes IFN-β production via the cGAS-STING-TBK1 pathway.

### The activated NLRP3 and NLRC4 inflammasomes inhibit the production of IFN-β.

**FIG 3:**
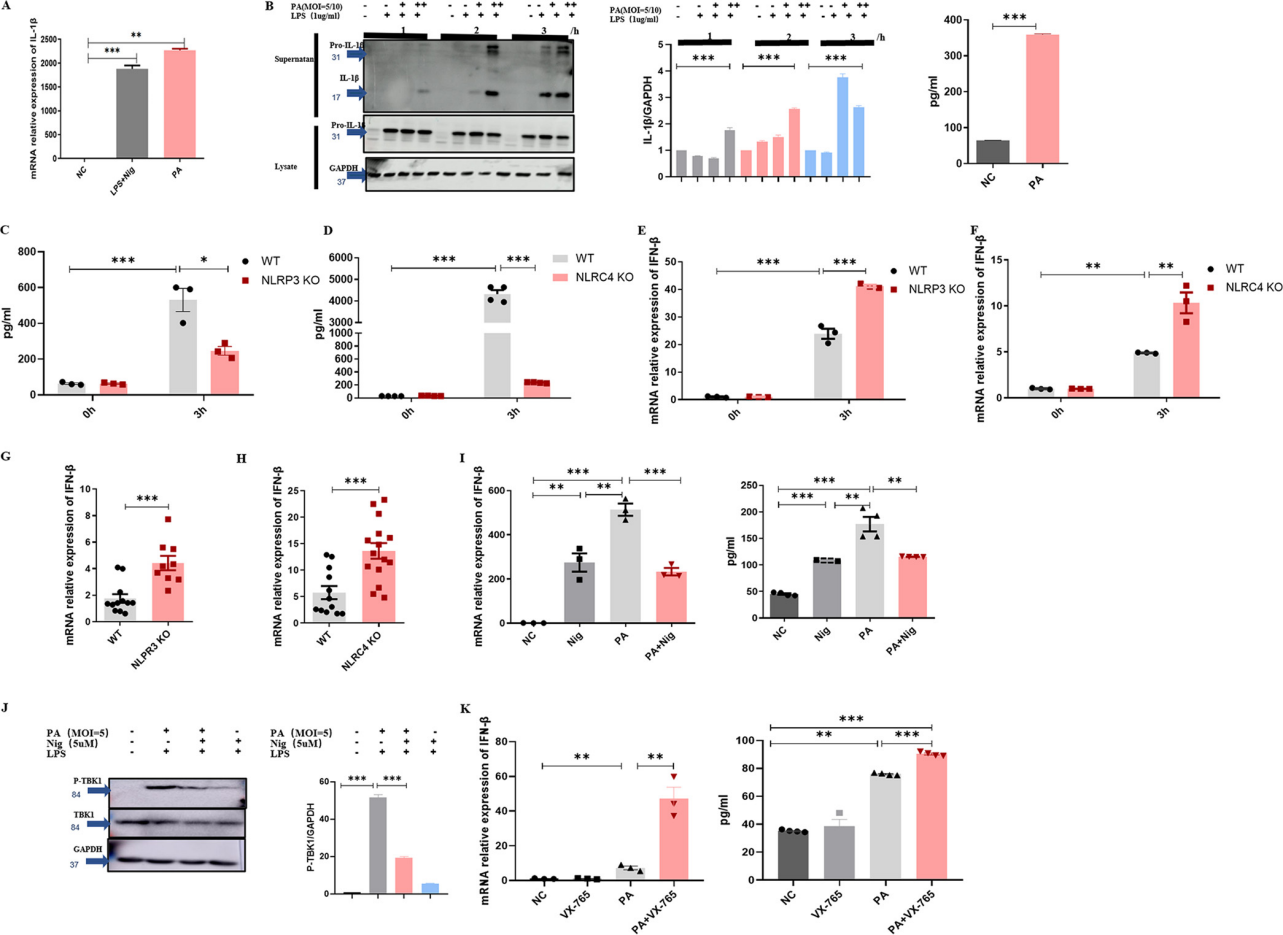
The activated NLRP3 and NLRC4 inflammasomes inhibit the production of IFN-β. (A) The mRNA relative expression of IL-1β in immortalized bone marrow-derived macrophages (iBMDMs) with PA (MOI = 10) infection for 1 h (*n* ≥ 3). (B) Immunoblotting for pro-interleukin-1β (pro-IL-1β), IL-1β, and GAPDH of iBMDM with PA (MOI = 5/10) infection for indicated time; the level of IL-1β in mouse peritoneal macrophages supernatant for PA (MOI = 5) infection for 2 h (*n* ≥ 3). (C) The level of IL-1β in WT and NLRP3 KO mouse peritoneal macrophages supernatant for PA (MOI = 1) infection for indicated time (*n* ≥ 3). (D) The level of IL-1β in WT and NLRC4 KO mouse peritoneal macrophages supernatant for PA (MOI = 1) infection for indicated time (*n* ≥ 3). (E) The mRNA relative expression of IFN-β in WT and NLRP3 KO mouse peritoneal macrophages with PA (MOI = 1) infection for indicated time (*n* ≥ 3). (F) The mRNA relative expression of IFN-β in WT and NLRC4 KO mouse peritoneal macrophages with PA (MOI = 1) infection for indicated time (*n* ≥ 3). (G) The mRNA relative expression of IFN-β in WT and NLRP3 KO mouse lung with PA (3 × 10^6^ CFU) infection for 24 h (*n* ≥ 3). (H) The mRNA relative expression of IFN-β in WT and NLRC4 KO mouse lung with PA (3 × 10^6^ CFU) infection for 24 h (*n* ≥ 3). (I) Pretreatment with Nig (5 μM) or DMSO as a control for 2 h; the mRNA relative expression of IFN-β in mouse peritoneal macrophages with PA (MOI = 1) infection for 3 h (*n* ≥ 3), pretreatment with Nig (5 μM) or DMSO as a control for 2 h, and the level of IFN-β of the mouse peritoneal macrophages supernatant with PA (MOI = 1) infection for 1 h (*n* ≥ 3). (J) Pretreatment with Nig (5 μM) or DMSO as a control for 2 h, immunoblotting for total TBK1, P-TBK1, and GAPDH in mouse peritoneal macrophages with PA (MOI = 5) infection for 1 h (*n* ≥ 3). (K) Pretreatment with VX-765 (10 μM) or DMSO as a control for 3 h, the mRNA expression of IFN-β in mouse peritoneal macrophages, and the level of macrophages supernatant with PA (MOI = 1) infection for 2 h (*n* ≥ 3). All experiments were performed three times. *, *P < *0.05; **, *P < *0.01; ***, *P < *0.001; ****, *P < *0.0001; LPS, lipopolysaccharide; Nig, nigericin.

Previous study has reported that NLRP4 negatively regulates type I interferon signaling by targeting TBK1 for degradation via E3 ubiquitin ligase DTX4 (28), and another study proved that inflammasome activation negatively regulated MyD88-IRF7 type I IFN signaling (29). Therefore, we focused attention on the inflammasome. NLRP3 and NLRC4 inflammasome play important roles in producing IL-1β. Consistent with previous studies, infection with PA and the mRNA expression of inflammasome protein complex were related to IL-1β elevated in the PA group (Fig. 3A). In addition, IL-1β in the cell supernatant was detected by Western blotting and enzyme-linked immunosorbent assay (ELISA) (Fig. 3B). In order to verify the critical role of the NLRP3 and NLRC4 inflammasome in producing IL-1β, we detected the level of IL-1β in the WT, NLRP3 KO, and NLRC4 KO mouse peritoneal macrophages supernatant (Fig. 3C and D). Obviously, IL-1β was decreased in the NLRP3 KO and NLRC4 KO group. We used the peritoneal macrophages from NLRP3 KO and NLRC4 KO mice (Fig. S1A, S1B) for an in-depth study. Fig. 3E and F and Fig. S2A and C showed that IFN-β interferon was enhanced in the NLRP3 KO and NLRC4 KO groups. To solidify our above observation, we tested the IFN-β expression in lung tissue of WT, NLRP3 KO, and NLRC4 KO mice after PA pulmonary infection for 24 h (Fig. 3G and H), and the results were consistent with cell experiments. In addition, inflammasome agonist nigericin (Nig) (Fig. S2E) inhibited IFN-β expression and TBK1 phosphorylation caused by PA infection (Fig. 3I and J), and caspase-1 inhibitor VX-765 (Fig. S2F) increased IFN-β expression after PA infection (Fig. 3K). The results *in vitro* and *in vivo* both demonstrated that the inhibition of NLRP3 and NLRC4 inflammasome could strengthen IFN-β production. To explore whether this regulation is general or dependent on PA infection, we conducted a series of supplementary experiments. HSV-1 and DNA extracted from PA instead of PA were used to infect mouse peritoneal macrophages; the inhibition of NLRP3 and NLRC4 inflammasome could weaken IFN-β production (Fig. S3A to C). The opposite phenomenon proved that this regulation was dependent on PA infection and should be noticed.

### IL-1β downstream of NLRP3 and NLRC4 inflammasome suppresses IFN-β production.

**FIG 4:**
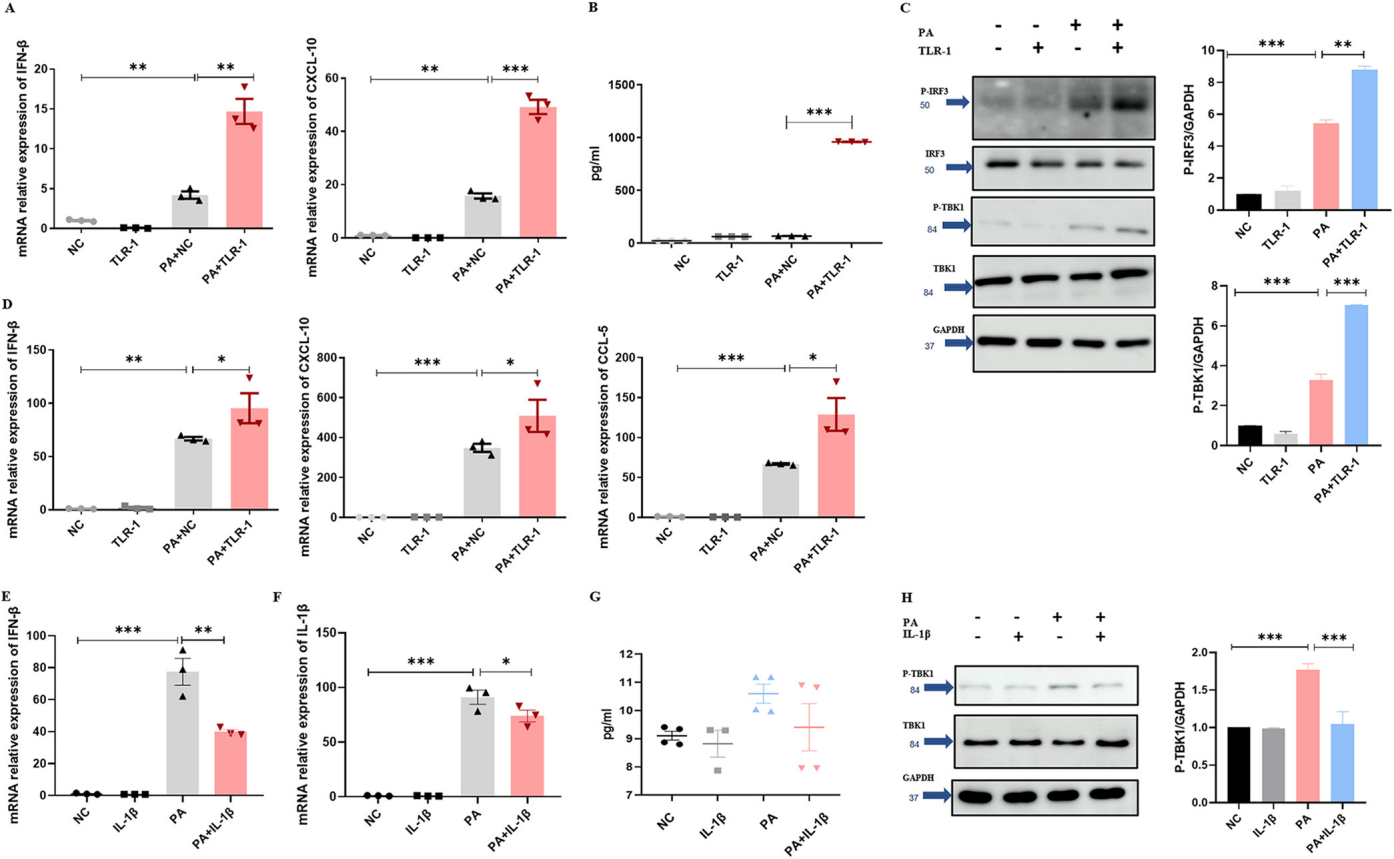
IL-1β downstream of NLRP3 and NLRC4 inflammasome suppressed IFN-β production. (A) Pretreatment with TLR-1 (33 μM) or ethanol as a control for 3 h; the mRNA expression of IFN-β and CXCL-10 in iBMDM with PA (MOI = 1) infection for 3 h (*n* ≥ 3). (B) Pretreatment with TLR-1 (33 μM) or ethanol as a control for 3 h; the level of IFN-β in iBMDM supernatant for PA (MOI = 1) infection for 3 h (*n* ≥ 3). (C) Pretreatment with TLR-1 (33 μM) or ethanol as a control for 3 h; immunoblotting for total TBK1, P-TBK1, total IRF3, and P-IRF3 and GAPDH in iBMDM with PA (MOI = 2) infection for 1 h. (D) Pretreatment with TLR-1 (33 μM) or ethanol as a control for 3 h; the mRNA expression of IFN-β, CXCL-10, and CCL-5 in mouse peritoneal macrophages with PA (MOI = 1) infection for 3 h (*n* ≥ 3). (E) Pretreatment with IL-1β (10 ng/mL) or PBS as a control for 3 h; the mRNA relative expression of IFN-β in mouse peritoneal macrophages with PA (MOI = 1) infection for 1 h (*n* ≥ 3). (F) Pretreatment with IL-1β (10 ng/mL) or PBS as a control for 3 h; the mRNA relative expression of IL-1β in mouse peritoneal macrophages with PA (MOI = 1) infection for 1 h (*n* ≥ 3). (G) Pretreatment with IL-1β (10 ng/mL) or PBS as a control for 3 h; the level of IFN-β in mouse peritoneal macrophages supernatant with PA (MOI = 1) infection for 1 h (*n* ≥ 3). (H) Pretreatment with IL-1β (20 ng/mL) or PBS as a control for 3 h; immunoblotting for total TBK1 and P-TBK1 and GAPDH in MH-S cells with PA (MOI = 2) infection for 1 h. All experiments were performed three times. *, *P < *0.05; **, *P < *0.01; ***, *P < *0.001; ****, *P < *0.0001.

Considering that many studies have mentioned that NLRP3, NLRC4, and STING-TBK1 were related, we tried coimmunoprecipitation and immunoblot analyses. Fig. S4A showed that NLRP3 or NLRC4 did not interacted with cGAS, STING, TBK1, or IRF3. IL-1β is an important effector downstream of NLRP3 and NLRC4 inflammasome. In this case, we speculated that IL-1β may have an association with cGAS-STING-TBK1-IFN-β. To verify our hypothesis, we tested IFN-β expression with the pretreatment of Toll-like receptor-1 (TLR-1; IL-1 receptor1 inhibitor) and synthetic mouse IL-1β protein. Fig. 4A, B, and D displayed the augmented IFN-β production and CXCL-10 expression in response to PA with TLR-1 pretreatment, and TBK1 phosphorylation and IRF3 phosphorylation were enhanced in the PA + TLR-1 group (Fig. 4C). Moreover, the synthetic mouse IL-1β protein reduced IFN-β production (Fig. 4E and G) and TBK1 phosphorylation induced by PA (Fig. 4H). However, the synthetic mouse IL-1β protein also reduced IL-1β (Fig. 4F) production in response to PA, which may be explained by the feedback inhibition. In addition to IL-1β, gasdermin D (GSDMD), which has pore-forming activity, is also downstream of the NLRP3 and NLRC4 inflammasome. GSDMD inhibitor disulfiram inhibited IFN-β and CXCL-10 expression (Fig. S5A and B) and IRF3 phosphorylation (Fig. S5C).

### IL-1β reduces the production of cGAMP and IFN-β by promoting AKT phosphorylation.

**FIG 5:**
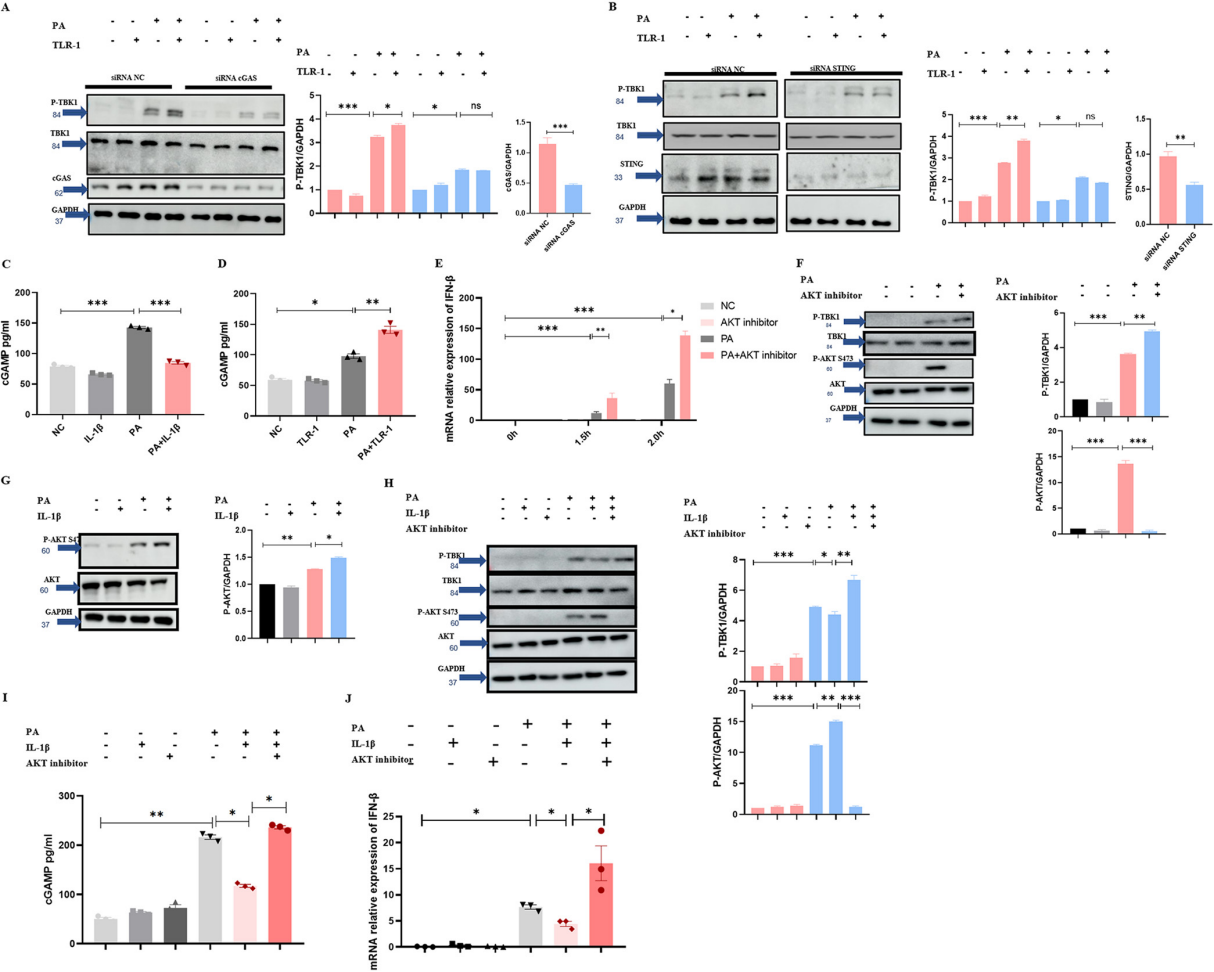
IL-1β inhibited cGAS-STING-TBK1-IFN-β axis by reducing the production of cGAMP. (A) Transfection with siRNA cGAS, immunoblotting for total TBK1, P-TBK1, total IRF3, cGAS, and GAPDH of MH-S with PA (MOI = 2) infection for 1 h. (B) Transfection with siRNA STING; immunoblotting for total TBK1, P-TBK1, total IRF3, STING, and GAPDH of MH-S with PA (MOI = 2) infection for 1 h. (C) Pretreatment with IL-1β (20 ng/mL) or PBS as a control for 3 h; the level of cGAMP in RAW264.7cells with PA (MOI = 5) infection for 45 min (*n* ≥ 3). (D) Pretreatment with TLR-1 (33 μM) or ethanol as a control for 3 h; the level of cGAMP in RAW264.7cells with PA (MOI = 5) infection for 45 min (*n* ≥ 3). (E) Pretreatment with AKT inhibitor (10 μM) or DMSO as a control for 3 h; the mRNA relative expression of IFN-β in RAW264.7 cells with PA (MOI = 5) infection for indicated time (*n* ≥ 3). (F) Pretreatment with AKT inhibitor (10 μM) or DMSO as a control for 3 h; immunoblotting for total AKT, P-AKT, total TBK1, P-TBK1, and GAPDH in RAW264.7 cells with PA (MOI = 5) infection for 45 min. (G) Pretreatment with IL-1β (20 ng/mL) or PBS as a control for 3 h; immunoblotting for total AKT, P-AKT, and GAPDH in RAW264.7 cells with PA (MOI = 5) infection for 45 min. (H) Pretreatment with IL-1β (20 ng/mL) and AKT inhibitor (10 μM) or PBS and DMSO as a control for 3 h; immunoblotting for total AKT, P-AKT, total TBK1, P-TBK1, and GAPDH in RAW264.7 cells with PA (MOI = 5) infection for 45 min. (I) Pretreatment with IL-1β (20 ng/mL) and AKT inhibitor (10 μM) or PBS and DMSO as a control for 3 h; the level of cGAMP in RAW264.7 cells with PA (MOI = 5) infection for 1 h (*n* ≥ 3). (J) Pretreatment with IL-1β (20 ng/mL) and AKT inhibitor (10 μM) or PBS and DMSO as a control for 3 h; the mRNA relative expression of IFN-β in RAW264.7 cells with PA (MOI = 5) infection for 1 h (*n* ≥ 3). All experiments were performed three times. *, *P < *0.05; **, *P < *0.01; ***, *P < *0.001; ****, *P < *0.0001.

Above all, PA causes IFN-β production via the cGAS-STING-TBK1 axis. Whether the inhibitory effect of IL-1β on IFN-β production was dependent on the cGAS-STING-TBK1 axis was still unclear. cGAS small interfering RNA (siRNA) and STING siRNA were used to reduce cGAS and STING expression. We found that the enhanced TBK1 phosphorylation in TLR-1 + PA group disappeared in the cGAS siRNA and STING siRNA cells (Fig. 5A and B). We next sought to define the mechanism involved in the IFN-β suppressive function from IL-1β. We found that the cellular quantity of cGAMP in the group of PA + IL-1β is lower than the group of PA (Fig. 5C), and TLR-1 augments the level of the cellular quantity of cGAMP (Fig. 5D). Taken together, these data suggested that IL-1β reduced PA-induced IFN-β production by suppressing cGAMP production. Considering that a study has suggested that AKT phosphorylated the S291 of the enzymatic domain of mouse cGAS and that AKT-mediated phosphorylation robustly suppresses cGAS enzymatic activity, which reduced the production of cGAMP and IFN-β (30). Based on that, we tested the expression of IFN-β and TBK1 phosphorylation after the pretreatment with AKT inhibitor VIII, which can inhibit the phosphorylation of AKT S473. Fig. 5E and F shows that AKT inhibitor VIII promoted IFN-β expression and TBK1 phosphorylation. Subsequently, IL-1β was found to promote AKT phosphorylation at S473 (Fig. 5G and H). Importantly, the reduction of TBK1 phosphorylation, cGAMP and IFN-β caused by IL-1β was reversed by the addition of AKT inhibitor VIII (Fig. 5H to J). Therefore, we concluded that IL-1β inhibits cGAS activity and reduces cGAMP and IFN-β production by promoting AKT phosphorylation in the process of PA infection.

### Lung injury and bacterial load reduced in IFN-β KO mouse after PA pulmonary infection.

**FIG 6:**
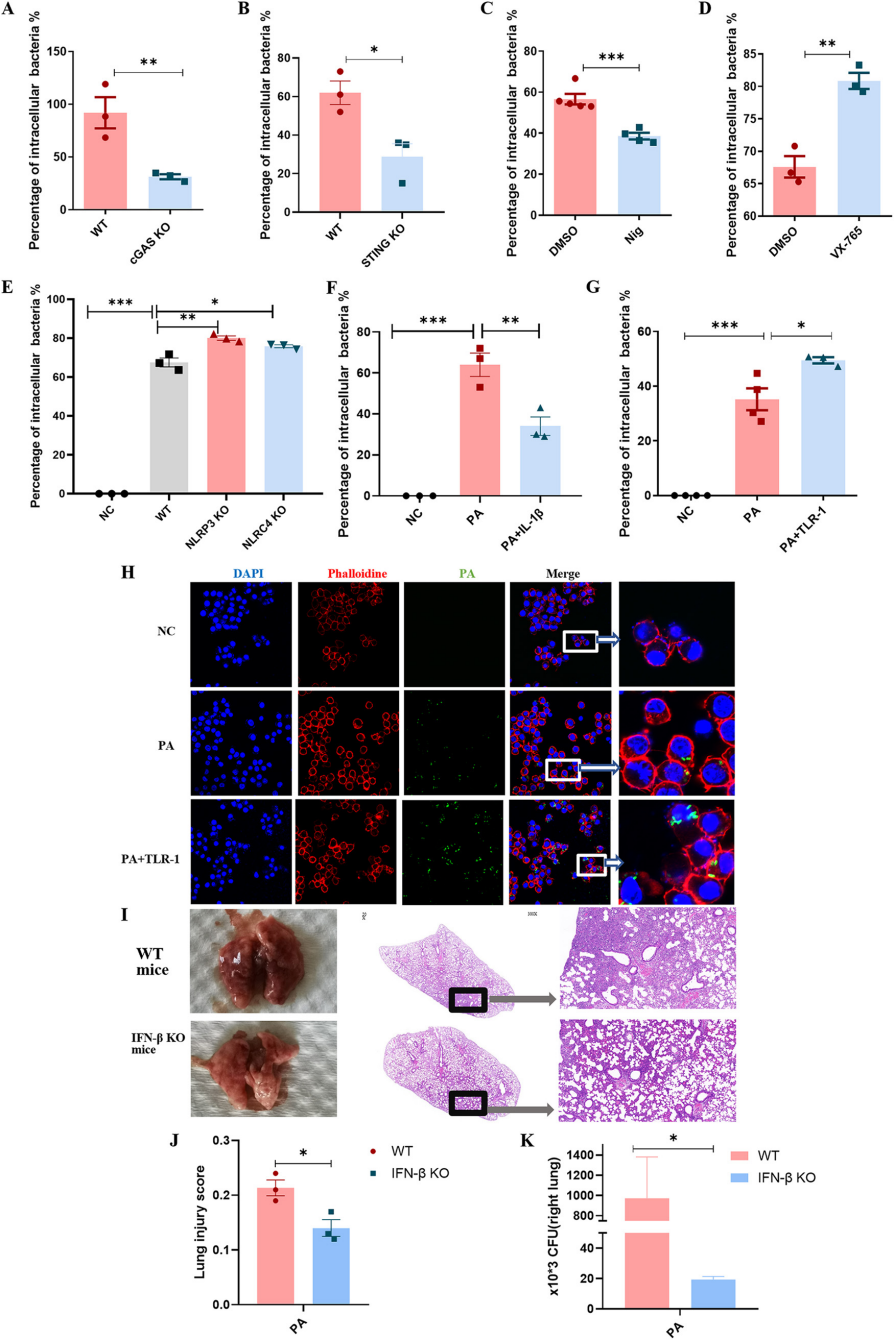
IFN-β promoted the survival of intracellular PA in macrophages. (A) The percentage of intracellular PA of WT and cGAS KO mouse peritoneal macrophages with PA (MOI = 0.1) infection for 2 h (*n* ≥ 3). (B) The percentage of intracellular PA of WT and STING KO mouse peritoneal macrophages with PA (MOI = 0.1) infection for 2 h (*n* ≥ 3). (C) Pretreatment with Nig (5 μM) or DMSO as a control for 2 h; the percentage of intracellular PA of mouse peritoneal macrophages with PA (MOI = 0.1) infection for 1 h (*n* ≥ 3). (D) Pretreatment with VX-765 (10 μM) or DMSO as a control for 3 h; the percentage of intracellular PA of mouse peritoneal macrophages with PA (MOI = 0.1) infection for 1 h (*n* ≥ 3). (E) The percentage of intracellular PA in WT, NLRP3 KO, and NLRC4 KO mouse peritoneal macrophages with PA (MOI = 0.1) infection for 1 h (*n* ≥ 3). (F) Pretreatment with IL-1β (10 ng/mL) or ethanol as a control for 3 h; the percentage of intracellular PA in RAW264.7 cells with PA (MOI = 0.1) infection for 1 h (*n* ≥ 3). (G) Pretreatment with TLR-1 (33 μM) or ethanol as a control for 3 h; the percentage of intracellular PA in RAW264.7 cells with PA (MOI = 0.1) infection for 1 h (*n* ≥ 3). (H) Pretreatment with TLR-1 (33 μM) or ethanol as a control for 3 h; the intracellular PA in RAW264.7 cells with GFP-labeled PA (MOI = 5) infection for 2 h. (I) Representation appearance of lungs from WT and IFN-β KO mice with PA (3 × 10^6^ CFU) infection for 24 h. Hematoxylin and eosin (H&E) staining of lungs from WT and IFN-β KO mice with PA (3 × 10^6^ CFU) infection for 24 h. (J) The lung injury score of WT and IFN-β KO mice right lung with PA (3 × 10^6^ CFU) infection for 24 h (*n* ≥ 3). (K) The bacterial CFU of WT and IFN-β KO mice right lung with PA (3 × 10^6^ CFU) infection for 24 h (*n* ≥ 3). All experiments were performed three times. *, *P < *0.05; **, *P < *0.01; ***, *P < *0.001; ****, *P < *0.0001.

IFN-β is one of the vital ingredients in anti-tumor, anti-virus, and antituberculosis immunity, but the effect of IFN-β on the survival of intracellular PA has not been studied. First, we carried out antibacterial experiments and confirmed that the addition of exogenous synthetic IFN-β had no obvious promotion or inhibition effect on PA growth (Fig. S6A and B), whereas the decreased survival of PA in the cGAS KO cells and STING KO cells caught our attention (Fig. 6A and B). Fig. 2C and D showed a drop of IFN-β in the cGAS KO cells and STING KO cells. We next tested the percentages of intracellular PA with pretreatment of Nig and VX-765. Nig reduced PA intracellular survival (Fig. 6C), and VX-765 increased intracellular survival (Fig. 6D). At the same time, the silence of NLRP3 and NLRC4 (Fig. 6E) did increase the survival of intracellular PA, respectively. Fig. 6F indicated that the addition of IL-1β can reduce PA survival. For more, TLR-1 promoted PA survival in the cytoplasm (Fig. 6G and H). For validating the effect of IFN-β *in vivo*, we constructed a series of experiments on animals. *In vivo*, the results showed that hemorrhage and edema of lung tissue were improved in IFN-β KO mice compared to in wild-type mice (Fig. 6I). At the same time, the lung injury scores and CFU of right lungs were reduced (Fig. 6J and K).

## DISCUSSION

Since PA was previously known as an extracellular bacterium, there were few reports on intracellular PA. The intracellular PA could eventually induce macrophages cell lysis, and the T3SS mutant exhibited defects in phagosomal escape and macrophage lysis driven by internalized bacteria (8). Similarly, an earlier study suggested that the T3SS allows PA to evade lysosomal killing within corneal epithelial cells after invasion, and PA can avoid lysosomal killing by surviving/replicating within intracellular vacuoles (7). Others found that epithelial cells developed pod-like clusters of intracellular PA with regional variation in protein expression (5). Importantly, compared to bacterial culture under planktonic conditions, the intracellular PA was insensitive to growth inhibition or killing by antibiotics that were capable of intraepithelial cell penetration, which can contribute to development of chronic infection (5). Significantly, a study identified class II ribonucleotide reductase enzyme (RNR) as the enzyme that supplies dNTPs to intracellular PA. This discovery could contribute to the development of RNR-targeted strategies against the chronicity occurring in this type of lung infection (31). Unlike the above, IFN-β was found to promote the survival of PA in the cytoplasm from our data. Considering that type I IFN-associated suppression of type 17 immunity and antimicrobial peptide production served as a conserved mechanism for enhanced susceptibility to both E. coli and S. aureus coinfection (19), we explored the relationship between IFN-β and antimicrobial peptide, but the results was negative (Fig. S7). Another suggestion is that abundant type I IFN predisposes lymphocytes to apoptosis, resulting in suppression of innate responses via increased IL-10 (32). From the observation that type I IFN stimulates production of IL-27, a cytokine that strongly suppresses IL-17A production (33), we suspect that the mechanism by which IFN can promote the survival of intracellular PA may be related to the immune factors of the host like phagocytosis of bacteria by lysosomes or the virulence factor of PA, but this needs to be further explored.

The association between inflammasome and cGAS-STING-TBK1 axis is intricate. One study stated that NLRP4 recruits the E3 ubiquitin ligase DTX4 to TBK1 for Lys48 (K48)-linked polyubiquitination, which led to degradation of TBK1, and plays a negative role in IFN-β production (28). However, Fig. S4A shows that NLRP3 or NLRC4 did not interacted with cGAS, STING, TBK1, or IRF3. Another study demonstrated that caspase-1 interacts with cGAS, cleaving it and dampening cGAS-STING-mediated IFN production (34). Based on the above research, we repeated the experiments with Nig, and it did not result in a reduction in cGAS protein or its cleavage (Fig. S4B). Aarreberg et al. claimed that IL-1β treatment results in IFN production and activation of IFN signaling to direct a potent innate immune response that restricts dengue virus infection (35). It is interesting that this finding was different from our conclusion (Fig. 4G and H). This may be due to the fact that our results were based on PA infection and not dengue virus infection. The research of Banerjee et al. suggests that GSDMD depletes intracellular potassium (K^+^) via membrane pores, and this K^+^ efflux was necessary to inhibit cGAS-dependent IFN-β response in the infection of DNA virus (36). This means that GSDMD retrained IFN-β response by disrupting ionic homeostasis. However, in our PA stimulation model, the production of IFN-β decreased after the use of GSDMD inhibitors, which was inconsistent with the results of Banerjee et al. (36). We thought carefully about the opposite effects of IL-1β receptor inhibitor TLR-1 and GSDMD inhibitor disulfiram on IFN-β response. GSDMD is known be involved in pyroptosis, which is defined as GSDM-mediated programmed death (37). The GSDM superfamily is composed of GSDMA/B/C/D, GSDME, and DFNB59; GSDMA/B/C/D consists of two conserved domains: the N-terminal pore-forming domain and the C-terminal repressor domain (38–40). In addition to GSDMD, other proteins in the family can also cause pore formation. GSDMC and GSDME are specifically cleaved by caspase-8 and caspase-3, generating an N-terminal domain that forms pores on cell membrane for inducing pyroptosis (41, 42). In Fig. S5, disulfiram is a specific inhibitor of GSDMD, which could inhibit only GSDMD pore formation and could not inhibit the effects of other proteins in the GSDM superfamily. Thus, the effects of other proteins may be compensatory to enhance due to the decrease of GSDMD. The relationship between IFN-β and pyroptotic cell death needs further research; for example, to determine the construction of GSDM protein family gene knockout mice.

Type I IFNs are induced by most, if not all, bacterial pathogens. Bacterial induction of type I IFNs occurs upon stimulation of two main pathways: first, Toll-like receptor (TLR) recognition of bacterial molecules such as lipopolysaccharide (LPS); and second, extracellular or phagosomal bacteria utilizing secretion systems, which can leak or secrete nucleic acids that are sensed via cytosolic pathways (26). Recently, a finding identified cGAS as a DNA sensor for detecting and restricting extracellular pathogen PA by promoting the production of type I IFNs (43). The results of earlier research also reflected that the cGAS-STING-IFN-β axis is critical for host defense in extracellular pathogens, supporting the role of type I IFNs in restricting the attack of PA infection (44, 45). In contrast, another study demonstrated that type III interferons lead to lethal bacterial superinfection for inducing barrier damage (46). Our research suggested for the first time that IFN-β is beneficial to the survival of intracellular PA. The production of IFN-β is inextricably related to IL-1β-AKT pathway. In the PA infection, this has never been described before. It forced us to rethink the clinical approach to control chronic pulmonary PA infection.

### Conclusion.

**FIG 7:**
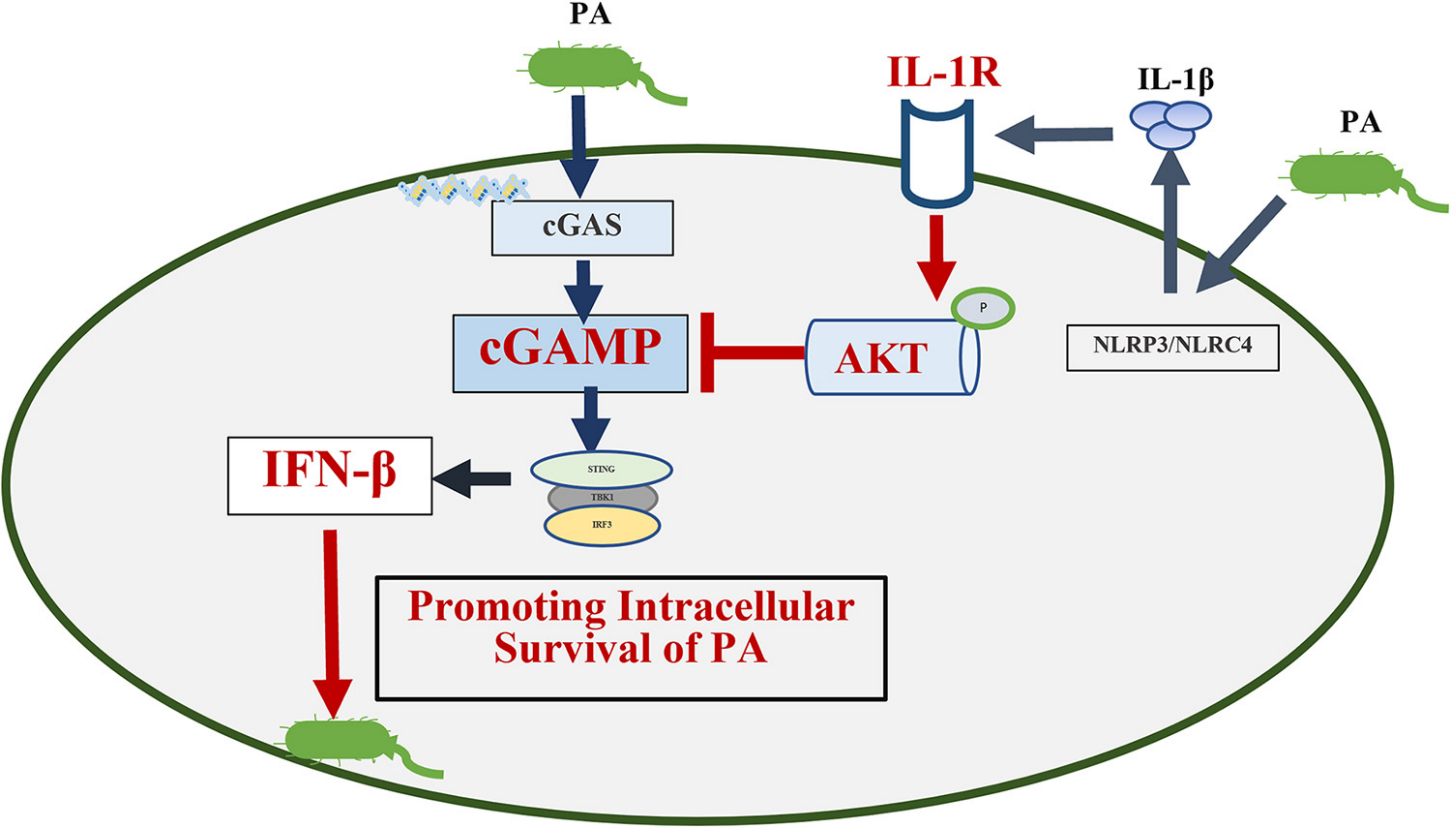
Graphical abstract of the research. PA triggered IFN-β production to promote its intracellular through cGAS-STING-TBK1 pathway. For host defense, IL-1β secreted by macrophages induced by PA suppress IFN-β response driven by cGAS-STING-TBK1 pathway. Mechanistically, IL-1β promoted AKT phosphorylation by binding to IL-1 receptors on the cell membrane. Phosphorylated AKT reduced the production of GAMP and inhibited the cGAS-STING-TBK1 signaling pathway, resulting in a decrease in the production of IFN-β. cGAMP, cyclic GMP-AMP; cGAS, cGAMP synthase.

In summary, our study illuminated the role of the IFN-β in deteriorating intracellular PA infection and confirmed that synthetic IFN-β enhanced the intracellular survival of PA. Furthermore, IL-1β not only acts as a signaling molecule needed for the production of inflammatory cytokines; our experimental evidence also indicated that the IL-1β is a negative regulator of IFN-β production in particular PA infection. Specifically, IL-1β activated AKT kinase, and expression of activated AKT led to the reduction of cGAMP production. Finally, the reduced cGAMP decreased IFN-β expression. Collectively, this work establishes a novel module involving IL-1β restricted IFN-β production during PA infection (Fig. 7). The inhibition of IFN-β may be used as a potential therapeutic method against pulmonary PA infection.

## MATERIALS AND METHODS

### Animals and bacteria.

All C57/B6 mice weighing 16 to 18 g were from Shanghai SLAC Laboratory Animal Co., Ltd. (China). NLRP3 KO and NLRC4 KO mice (C57/B6 background) were housed in specific pathogen-free (SPF) conditions within an animal care facility (Center of Laboratory Animal, Tongji University, Shanghai, China) until euthanization. All animals were cared for in accordance with National Institutes of Health guidelines, and all animal experiments were performed under the guidance of and with approval from the Institutional Animal Care and Use Committee of Tongji University (approval no. TJBE00120101). P. aeruginosa (ATCC-BAA-47; strain HER-1018/PAO1) was used in these experiments.

### Murine model of PA lung infection.

A pulmonary PA infection model was established in the following way. PA and 6- to 8-week-old SPF C57BL/6 female and NLRP3 KO and NLRC4 KO mice (C57BL/6 background) were used to establish the murine model of PA lung infection. Briefly, bacterial concentrations were validated by plating on Luria Broth (LB) agar and counting CFU. Before each experiment, the bacterial solution was washed twice and resuspended in phosphate-buffered saline (PBS). After being anesthetized with isoflurane, the mice were infected intratracheally with PA (3 × 10^6^ CFU) in 35 μL of PBS on day 1 (47). Then, 24 h postinfection, the mice were euthanized, and the lung samples were collected for further analysis.

### Cell culture and treatment.

The mouse peritoneal macrophages (MPMs) extracted from C57BL/6 female mice and MH-S cells and were cultured routinely in Roswell Park Memorial Institute (RPMI) medium (HyClone) supplemented with 10% fetal bovine serum (FBS), l-glutamine, and penicillin. Immortalized bone marrow-derived macrophage (iBMDM) cells and Raw264.7 cells were cultured routinely in Dulbecco’s modified Eagle’s medium (DMEM) (HyClone) supplemented with 10% FBS, l-glutamine, and penicillin. All cells were cultured in antibiotic-free medium before PA stimulation or inhibitor pretreatment. The cells were pretreated with VX-765 (HY-13205, Med Chem Express), Nig (HY-13205, Med Chem Express), IL-1β (HY-P7073, Med Chem Express), IL-1β receptor inhibitor (TLR-1, HY-W011400, Med Chem Express), AKT inhibitor VIII (HY-10355, Med Chem Express), or IFN-β (CAT 300-02BC, PeproTech) for the indicated times before stimulation with PA, as indicated; multiplicity of infection (MOI) values are given for the different times.

### The extracted of PA DNA.

We extracted DNA from logarithmic growth phase PA with PureLink microbiome DNA purification kit (2077129, Invitrogen) according to the manufacturer’s protocols.

### Percentages of intracellular PA.

All of the cells were cultured in antibiotic-free medium before PA infection for indicated hours. After the stimulation, the cells were washed three times with PBS and replaced with a new medium. The cells were treated with gentamicin (150 μg/mL) for 1 and 3 h. Next, 1% Triton X-100 was used to lyse the cells, the cell lysate was cultured on LB agar plate for 24 h at 37°C, and the CFU were counted. The calculation formula for the percentage of intracellular PA = (the number of CFU after 3 h of gentamicin treatment/the number of CFU after 1 h of gentamicin treatment) × 100%.

### ELISA.

Cell culture supernatants and mouse lung homogenates were collected for ELISA. IL-1β concentrations were measured by mouse IL-1β ELISA kits (Thermo Fisher) according to the manufacturer’s protocols. IFN-β concentrations were measured by mouse IFN-β ELISA kits (R&D) according to the manufacturer’s protocols. cGAMP concentrations were measured by mouse cGAMP ELISA kits (Arbor Assays) according to the manufacturer’s protocols.

### Confocal microscopy.

Cells were infected with GFP-labeled PA. The cells were washed with PBS, fixed with 4% paraformaldehyde, permeabilized with 0.1% Triton X-100, blocked by 1% bovine serum albumin (BSA), and then incubated with LC-3B (ab48394, Abcam) primary antibodies overnight, followed by Alexa Fluor 555-labeled IgG (A0453, Beyotime), or stained with tetramethyl rhodamine isothiocyanate (TRITC)-(Phalloidin) (100 nM) for 30 min in the dark, and stained with 4′,6-diamidino-2-phenylindole (DAPI) for 10 min, respectively. The cells were visualized using a laser scanning confocal microscope (Nikon, Japan).

### Bacteria quantification in lung homogenate.

The mice were euthanized 1 day after the instillation of bacteria. To generate the lung homogenate, 10 μL/mg PBS was added, and the lungs were homogenized under sterile conditions. The homogenate was serially diluted and cultured on LB plates for 24 h at 37°C; bacteria CFU were then counted.

Statistical analysis of data was performed using GraphPad Prism software 8.0. One-tailed unpaired Student’s *t* tests were used for analysis of different groups. Unless otherwise indicated, all experiments were performed at least three times and are presented as the mean ± standard error of mean (SEM). A *P* value of <0.05 was considered significant.

### Quantitative reverse transcription-PCR.

Total RNA was isolated using TRIzol (Thermo Fisher), and then 1 μg RNA was reverse transcribed using Prime Script RT Master Mix (Toyobo). Quantitative PCR was performed using the Power SYBR green PCR Master Mix (Toyobo). The amounts of transcript were normalized to those of glyceraldehyde-3-phosphate dehydrogenase (GAPDH).

### Primers.

The following primers were used: mouse GAPDH, up CCCACTAACATCAAATGGGG and down CCTTCCACAATGCCAAAGTT; mouse tumor necrosis α (TNF-α), up TTCTGTCTACTGAACTTCGGGGTGATCGGTCC and down GTATGAGATAGCAAATCGGCTGACGGTGTGGG; mouse IL-6, up TCCAGTTGCCTTCTTGGGAC and down GTGTAATTAAGCCTCCGACTTG; mouse IL-1β, up TCTTTGAAGTTGACGGACCC and down TGAGTGATACTGCCTGCCTG; mouse IFN-β, up CAGCTCCAAGAAAGGACGAAC and down GGCAGTGTAACTCTTCTGCAT; mouse CXCL-10, up CCAAGTGCTGCCGTCATTTT and down GATAGGCTCGCAGGGATGAT; and mouse CCL-5, up CCTCACCATATGGCTCGGAC and down TCTTCTCTGGGTTGGCACAC.

### Western blotting.

Cells or tissues were washed with cold PBS and lysed in radioimmunoprecipitation assay (RIPA) buffer containing protease inhibitors cocktail (Roche) followed by standard Western blotting procedure. After measuring protein concentration, the samples were loaded and separated on 10 to 12% precast polyacrylamide gels and then transferred to NC membranes (GE) at 100 V for 10 min. The membranes were blocked with 5% no-fat powdered milk and then incubated with primary antibodies overnight, followed by secondary antibodies. The specific signals were detected by Immobilon Western Chemiluminescent horseradish peroxidase (HRP) substrate (Millipore) and the Tanon image system. The following antibodies were used: GAPDH (5174, Cell Signaling Technology), cGAS (31659, Cell Signaling Technology), STING (13647, Cell Signaling Technology), TBK1 (3504, Cell Signaling Technology), P-TBK1 (5483, Cell Signaling Technology), IRF3 (4302, Cell Signaling Technology), P-IRF3 (4947, Cell Signaling Technology), IL-1β (5129, Biovision), AKT (4691, Cell Signaling Technology), P-AKT S473 (4060, Cell Signaling Technology), and HRP-conjugated anti-rabbit IgG (H+L) as a secondary antibody (Beyotime).

### Transfection of siRNA.

Before transfection, the cell culture medium was replaced with preheated serum-free medium. Then we added 50 μL serum-free medium and GP-transfect-Mate transfection reagent (G04009, Gene pharma) into a 1.5-mL sterile centrifuge tube, mixed well, and let it stand for 5 min. Similarly, we added 50 μL serum-free medium and RNA oligonucleotide into a 1.5-mL sterile centrifuge tube, mixed well, and let it stand for 5 min. The two mixtures were mixed evenly and left for 20 min before transfection. After transfection for 4 to 6 h, the culture medium was changed to completed medium. After transfection for 72 h, the transfection efficiency was tested by Western blotting with the following primers: siRNA cGAS, 5′-GGCUAGUGUGUCUAUUAAAUG-3′ and 3′-UUUAAUAGACACACUAGCCAU-5′; and siRNA STING, 5′-GGAUCCGAAUGUUCAAUCAGC-3′ and 3′-UGAUUGAACAUUCGGAUCCGG-5′.

### The identification of NLRP3 KO and NLRC4 KO mice.

NLRP3 KO and NLRC4 KO mice were identified using genotyping PCR for NLRP3 and NLRC4 gene expression, which was performed on tails that were obtained from mice aged 3 to 4 weeks old. PCR was performed using the following primers: NLRP3, GCTGTGCCCAGGTCCTAGC, CAGCAGCAGCCCTTTCGAG, GCAGCGCATCGCCTTCTATC, and CGGTGGTTGCTAGGAGATGG; and NLRC4, GAAGCCTCAACGGCAACGAGCACTC; GCAGGAATCAATCCAGAGTCTGAG, and GCAGCGCATCGCCTTCTATC. The PCR product produced by NLRP3 KO mouse was 275 bp long. The PCR product produced by NLRC4 KO mouse was 222 bp long. PCR-negative littermates and C57BL/6 mice were used as controls.

### Hematoxylin and eosin (H&E) staining.

At the appropriate time points, the mice were euthanized, and the diaphragm was carefully cut open without touching the lung. The entire lung was removed with surgical instruments, and the blood was washed away in PBS. Then, the lung was dissected and fixed in 4% formaldehyde at 4°C overnight before paraffin sectioning or cryosectioning. For cryosectioning, the fixed lung was settled by 30% sucrose before embedding into the Tissue-Tek O.C.T compound (Sakura) and solidified on dry ice, and the sample was cut into 5- to 10-μm-thick sections using a cryotome (Leica Microsystems). For paraffin sections, the lung was dehydrated by gradient ethanol in an automatic tissue processer (Leica Microsystems) and then embedded into paraffin blocks. The blocks were cut into 5- to 7-μm-thick sections using a microtome (Leica Microsystems, Germany) at distinct planes. The sections were placed on polylysine-coated glass slides and stored at room temperature for further use.

### Statistical analysis.

Statistical analysis of data was performed using GraphPad Prism software 8.0. One-tailed unpaired Student’s *t* tests were used for analysis of different groups. Unless otherwise indicated, all of the experiments were performed at least three times and are presented as the mean ± standard error of mean (SEM). A *P* value of <0.05 was considered significant.
