# Supplementary material for: Pseudomonas aeruginosa Induces Interferon-β Production to Promote Intracellular Survival
Source: Microbiol Spectr. 2022 Oct 3;10(5):e01550-22. doi: 10.1128/spectrum.01550-22 (PMC9603546; doi:10.1128/spectrum.01550-22)
Supplement: Supplemental file 1 — Fig. S1 to S7. Download spectrum.01550-22-s0001.pdf, PDF file, 0.6 MB [file spectrum.01550-22-s0001.pdf]

***Pseudomonas aeruginosa* induces Interferon-β production to promote intracellular survival**

Ling Yang¶[1,2], Yu-Wei Zhang¶[1,2], Yang Liu¶[1,2], Ying-Zhou Xie[2], Dong Weng[2], Bao-Xue Ge[3], Hai-Peng Liu[3], Jin-Fu Xu*[1,2]

1; Department of Respiratory and Critical Care Medicine, Shanghai Pulmonary Hospital, School of Medicine, Tongji University, Shanghai, China

2; Institute of Respiratory Medicine, School of Medicine, Tongji University, Shanghai, China

3; Clinical Translation Research Center, Shanghai Pulmonary Hospital, School of Medicine, Tongji University, Shanghai, China

*Corresponding author. E-mail: Jin-Fu Xu, MD, PhD, Department of Respiratory and Critical Care Medicine, Shanghai Pulmonary Hospital, School of Medicine, Tongji University. No. 507 Zhengmin Road, Shanghai 200433, China; E-mail: jfxu@tongji.edu.cn

¶These authors contributed equally to this work.

Supplementary figures

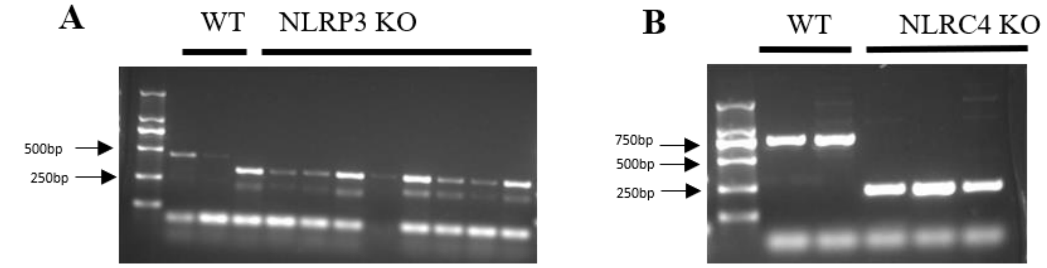

Fig S1; The identification of NLPR3 KO(A) and NLRC4 KO mouse(B).

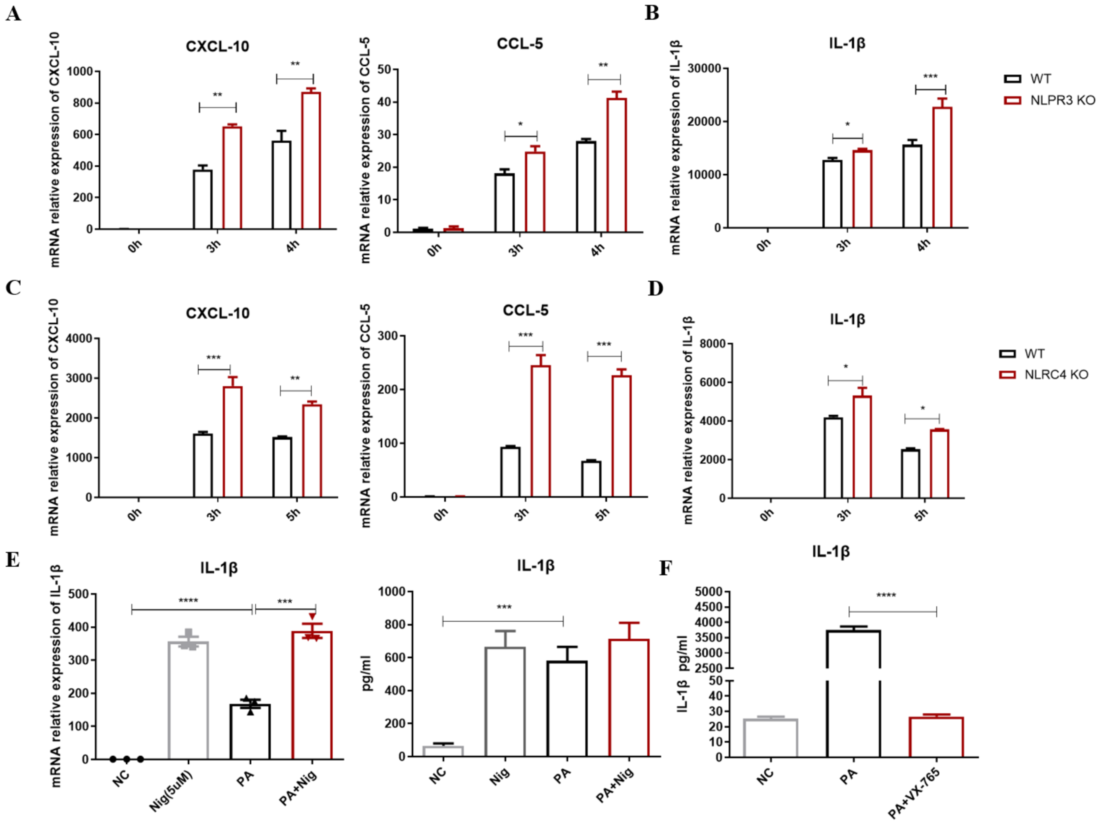

Fig S2; The activated NLRP3 and NLRC4 inflammasomes inhibit the production of IFN-β.

A; The mRNA relative expression of CXCL-10 and CCL-5 of WT and NLRP3 KO mouse peritoneal macrophages with PA (MOI=1) infection for indicated time, n⩾3.

B; The mRNA relative expression of IL-1β of WT and NLRP3 KO mouse peritoneal macrophages with PA (MOI=1) infection for indicated time, n⩾3.

C; The mRNA relative expression of CXCL-10 and CCL-5 of WT and NLRC4 KO mouse peritoneal macrophages with PA (MOI=1) infection for indicated time, n⩾3.

D; The mRNA relative expression of IL-1β of WT and NLRC4 KO mouse peritoneal macrophages with PA (MOI=1) infection for indicated time, n⩾3.

E; Pretreatment with Nig (5uM) or DMSO as a control for 2 hours, the expression of IL-1β of mouse peritoneal macrophages with PA (MOI=1) infection for 1hour, n⩾3.

F; Pretreatment with VX-765 (10uM) or DMSO as a control for 3 hours, the level of IL-1β of mouse peritoneal macrophages supernatant with PA (MOI=1) infection for 2 hour, n⩾3.

all experiments were performed three times, $*P < 0.05$; $**P < 0.01$; $***P < 0.001$; $****P < 0.0001$.

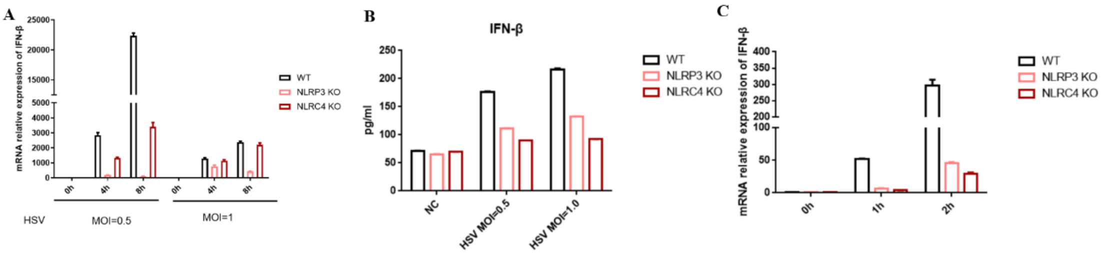

Fig S3; When stimulated with HSV and DNA extracted from PA, NLRP3 and NLRC4 inflammasomes promoted the production of IFN-β.

A; The mRNA relative expression of IFN-β of WT, NLRP3 KO and NLRC4 KO mouse peritoneal macrophages with HSV-1 infection for indicated time, n⩾3.

B; The level of IFN-β of WT, NLRP3 KO and NLRC4 KO mouse peritoneal macrophages supernatant with HSV-1 infection for 4 hours, n⩾3.

C; The mRNA relative expression of IFN-β of WT, NLRP3 KO and NLRC4 KO mouse peritoneal macrophages with DNA(2ug/ml) extracted from PA for indicated time, n⩾3.

all experiments were performed three times, *P < 0.05; **P < 0.01; ***P < 0.001; ****P < 0.0001.

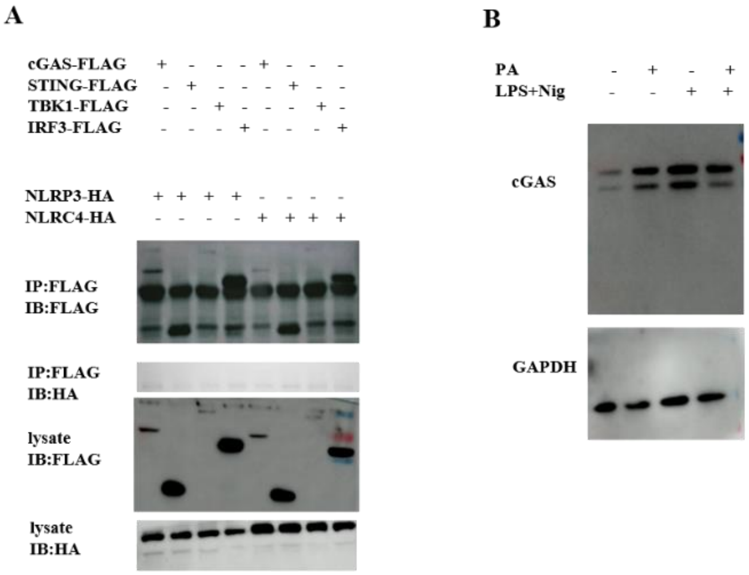

Fig S4; A; Coimmunoprecipitation and immunoblot analysis of 293FT cells transfected with various combinations (above lanes) of plasmid for Flag, Flag-cGAS, Flag-STING, Flag-TBK1, Flag-IRF3 and the HA- tagged NLRP3 or HA-tagged NLRC4.

B; Immunoblotting for cGAS and GAPDH in the lysates of mouse peritoneal macrophages stimulated with PA (MOI=5 for 1hour) or Nig (5uM).

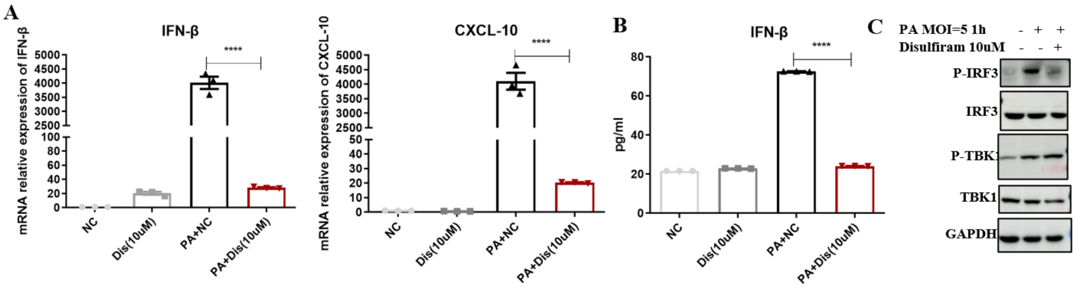

Fig S5: GSDMD inhibitor decreased the production of IFN-β.

A; Pretreatment with Disulfiram (10uM) or DMSO as a control, the mRNA relative expression of IFN-β and CXCL-10 of iBMDM with PA (MOI=1) infection for 3 hours, n⩾3.

B; Pretreatment with Disulfiram (10uM) or DMSO as a control for 3 hours, the level of IFN-β of iBMDM supernatant with PA (MOI=1) infection for 3 hours, n⩾3.

C; Pretreatment with Disulfiram (10uM) or DMSO as a control for 3 hours, immunoblotting for total TBK1, P-TBK1, total IRF3, P-IRF3 and GAPDH in the lysates of iBMDM with PA (MOI=5) infection for 1 hours.

all experiments were performed three times, *P < 0.05; **P < 0.01; ***P < 0.001; ****P < 0.0001.

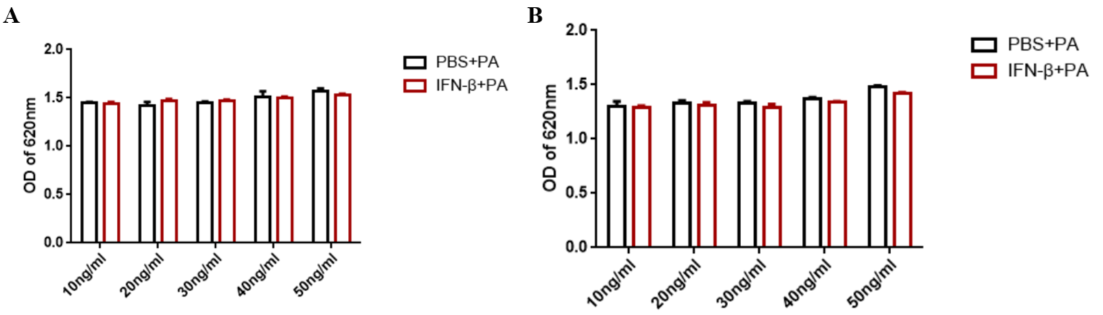

Fig S6; Exogenous supplementation of IFN-β had no significant effect on the growth of PA.

A 5x10^3CFU/ml, B 5x10^2CFU/ml.

all experiments were performed three times, *P < 0.05; **P < 0.01; ***P < 0.001; ****P < 0.0001.

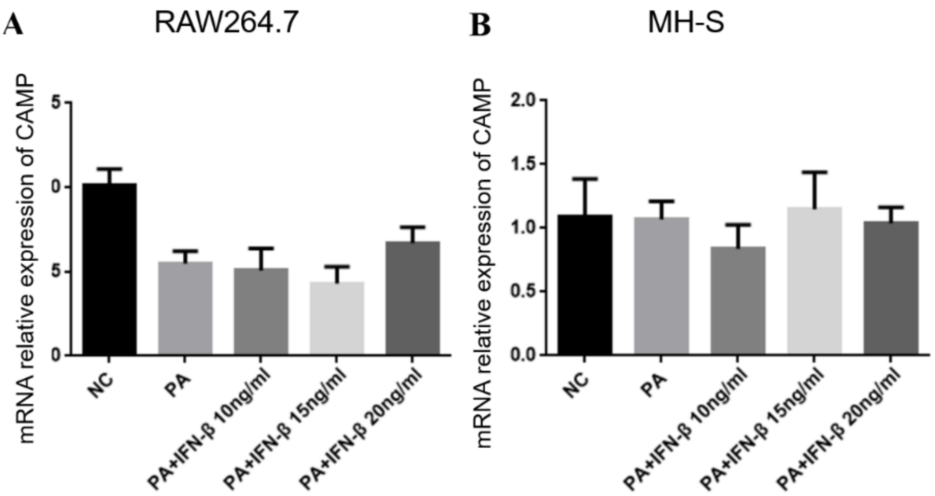

Fig S7; Pretreatment with IFN-β(10ng/ml, 15 ng/ml and 20ng/ml) or PBS as a control for 3 hours, the mRNA relative expression of CAMP of the RAW264.7 cells (A) and MH-S (B) with PA (MOI=1) infection for 4 hour, n≥3. *P < 0.05; **P < 0.01; ***P < 0.001; ****P < 0.0001.
